# Extended CT Void Analysis in FDM Additive Manufacturing Components

**DOI:** 10.3390/ma13173831

**Published:** 2020-08-30

**Authors:** Adriana Hernandez-Contreras, Leopoldo Ruiz-Huerta, Alberto Caballero-Ruiz, Verena Moock, Hector R. Siller

**Affiliations:** 1 Programa de Maestría y Doctorado en Ingeniería, Universidad Nacional Autónoma de México (UNAM), Building “S-Bernardo Quintana Arrioja”, 1st floor, University City, 04510 Mexico City, Mexico; adri.ihc@gmail.com; 2 Instituto de Ciencias Aplicadas y Tecnología, Universidad Nacional Autónoma de México, Circuito Exterior S/N, C. U., Delegación Coyoacán, 04510 Mexico City, Mexico; alberto.caballero@icat.unam.mx (A.C.-R.); verena.moock@icat.unam.mx (V.M.); 3 National Laboratory for Additive and Digital Manufacturing (MADiT), Mexico; 4 Department of Mechanical Engineering, University of North Texas, 3940 North Elm St., Denton, TX 76207, USA; hector.siller@unt.edu

**Keywords:** additive manufacturing (AM), digital manufacturing, void analysis, X-ray computed tomography (CT), mechanical properties

## Abstract

Additive manufacturing (AM) is the term for a number of processes for joining materials to build physical components from a digital 3D model. AM has multiple advantages over other construction techniques, such as freeform, customization, and waste reduction. However, AM components have been evaluated by destructive and non-destructive testing and have shown mechanical issues, such as reduced resistance, anisotropy and voids. The build direction affects the mechanical properties of the built part, including voids of different characteristics. The aim of this work is an extended analysis of void shape by means of X-ray computed tomography (CT) applied to fused deposition modeling (FDM) samples. Furthermore, a relation between the tensile mechanical properties and digital void measurements is established. The results of this work demonstrate that void characteristics such as quantity, size, sphericity and compactness show no obvious variations between the samples. However, the angle between the main void axis and the mechanical load axis α shows a relation for FDM components: when its mean value μ(α) is around 80 (degrees) the yield strength and Young’s modulus are reduced. These results lead to the formulation of a novel criterion that predicts the mechanical behavior of AM components.

## 1. Introduction

### 1.1. Additive Manufacturing

Additive manufacturing (AM) is defined by the standard ISO-ASTM 52900 [1] as “the process of joining materials to make parts from 3D model data, usually layer upon layer, as opposed to subtractive manufacturing and formative manufacturing methodologies”. According to that standard, AM is classified by seven general processes: material extrusion (ME), direct energy deposition (DED), material jetting (MJ), powder bed fusion (PBF), vat photopolymerization (VP), binder jetting (BJ), and sheet lamination (SL) [1]. From these processes and their combinations with others manufacturing processes, different technologies have been developed.

AM advantages include the possibility to manufacture freeform complex components that would otherwise be impossible to build with subtractive manufacturing processes, and product customization and personalization by reducing mass, amount of assemblies, and material waste in order to improve the supply chain operability [2,3,4,5,6]. However, AM has shown disadvantages: the presence of voids and inclusions due to the lack of material union or recrystallization [7]; mechanical anisotropy as a consequence of the layer-by-layer build characteristics; reduced resistance against external forces; surface inconsistencies, such as the stair-stepping effect and witness marks from support material removal; and residual stresses caused when the material is heated unevenly [5,6,8,9,10,11,12].

These issues have been evaluated and reported through various destructive testing characterization methodologies, including tensile, which has been widely used [11,12,13,14,15,16,17,18], fatigue, compression, bending, and impact [12,19,20,21]. These evaluations have demonstrated that AM components are weaker if a force or load is applied in the same build direction due to weak bonding and/or voids between the deposited materials, showing a laminated behavior, i.e., the components present anisotropy [11,12,13,15]. Studies based on evaluating density have shown lower tensile strength and anisotropy due to the presence of hollow voids in processes such as ME [16]. However, these voids are also present in other AM processes, such as DED and PBF, and lead to similar effects [18,22,23]. The characterization of voids in AM has also been carried out using different non-destructive testing (NDT) methodologies, such as X-ray computed tomography (CT) for particle and void analysis, as seen by du Plessis and others [23,24,25].

### 1.2. X-Ray Computed Tomography

CT is an NDT technique based on radiographic imaging [26,27,28]. For 3D inspection the object of interest is rotated 360 degrees while being irradiated by X-rays. Parameters such as the current in the filament, the accelerating voltage within the X-ray source, and exposure time are selected according to the material type and thickness to manage X-ray attenuation and to optimize images captured by a detector [29,30]. A 3D image is obtained by processing a stack of 2D projections using a reconstruction algorithm. Additional reconstruction features can be used to correct imaging artefacts such as beam hardening, X-ray scattering, and signal noise [30,31,32]. During postprocessing a grey level threshold can be selected in the reconstructed images to differentiate between materials within a sample and the background [30,33]. The estimation of an optimal threshold value is based on statistical methods and particular software intrinsic model assumptions that vary the results [33,34,35,36] with the consequence of errors like the partial volume effect [30,31,32].

CT is highly suitable for the inspection of defects, such as voids, cracks, and inclusions, at the micro, meso, and macro scale. The properties of voids are quantity, porosity percentage or void number; size measurements in different dimensions, such as the diameter or radius, area, and volume information about the circumscribing sphere that involves the void; spatial location inside the component; compactness, the ratio between the volume of the defect and the volume of the circumscribed sphere [37,38,39]; and sphericity, the ratio between the surface of a sphere with the same volume as the defect and the surface of the defect, which could be calculated by software [37,38,40].

### 1.3. Effect of Voids in Mechanical Properties

Voids are defects in the structure of components that modify their overall mechanical properties. Void generation occurs in the manufacturing process. In processes like metal casting, voids are caused by shrinkage, gas injection, and solidification time [41,42,43]; while for injected polymers the low injection pressure can cause the generation of bubbles [44]; and for composite materials, with a matrix of resin and polymer, the voids are caused by trapped air during the cure cycle [45,46]. In AM these defects are widely present due to trapped gas and the lack of bonding between model material [12,15,47,48].

The presence of voids in parts has an effect on tensile and fatigue strength [49,50,51,52]. High void content has been found to decrease the Young’s modulus, tensile strength, and hardness [52,53,54]. Voids in the interlayer bonding of composite materials cause delamination due to shear fracture [45,55,56]. However, it has been demonstrated that components with high void content have no correlation with the decrease of fatigue life [57] or on the debonding of material of AM components in tensile tests [58]. Furthermore, high void content results in a decrease in delamination of the composites [59]. Studies of void size, measured in 1, 2, and 3 dimensions, have shown that components with large voids have a decrease in their strength [52,60,61,62], while other results do not show an impact of void sizes with respect to fatigue test results [57]. The shape of voids measured by different descriptors, like sphericity and compactness, also express a variation of the Young’s modulus; spherical voids show less impact than those with elongated shape [52,55,60,63]. As a consequence of void shape, the orientation of large and elongated voids regarding the load direction has an impact on mechanical strength [25,50,64].

### 1.4. Aim and Scope

In AM, the void formation is common because the new material added during the build processes can lack bonding strength with respect to a previous layer. This phenomenon is present in all AM processes with different proportions and consequences [20,22,23]. Components manufactured by ME/FDM (fused deposition modeling) possess a higher amount of voids, since the addition of material is discrete and dependent on the filament shape and size [15,64,65]. Anisotropy is also a common characteristic of AM components, which is related to the build direction [13,14,17]. By using CT this work digitizes ME/FDM parts built in different orientations, in order to analyze the principal characteristics of elongated voids and their relation with tensile mechanical properties. Based on quantitative findings, a new descriptor that relates void characteristics with the mechanical properties, in particular their yield strength and Young’s modulus, is proposed.

### 1.5. ME/FDM Process

ME consists of material deposition, dispensed through a nozzle or orifice and deposited, usually heated, on a bed, layer upon layer [1,66]. The ME process is divided into several other technologies: FDM, fused filament fabrication (FFF), big area additive manufacturing (BAAM), direct ink writing (DIW), and bioprinting [64,66,67,68,69].

FDM technology has two different feedstock materials in the form of continuous filament contained within canisters. The first canister contains model material, which typically consists of thermoplastic polymers such as acrylonitrile butadiene styrene (ABS), nylon, polycarbonate (PC), or polylactic acid (PLA) [70]. The second canister material contains support material, which is used for cantilever sections of the component or to support holes. Support material must be removed after construction.

**Figure 1:**
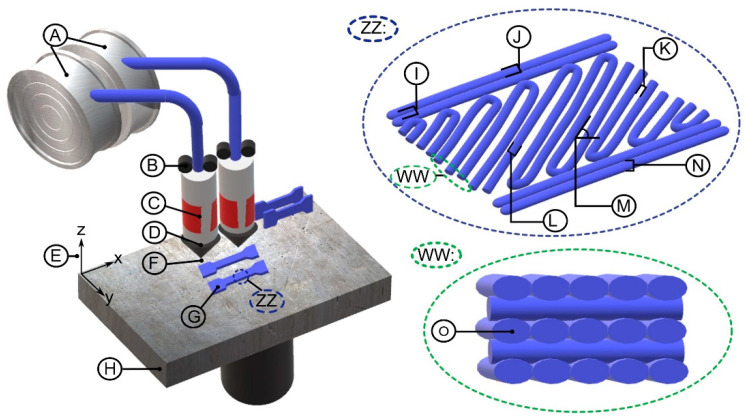
FDM (fused deposition modeling) technology principal components are represented by **A** material and support canisters, **B** rollers, **C** heating resistor, **D** nozzle, **E** build direction (*z*-axis), **F** nozzle tip, **G** component, and **H** building bed; FDM build parameters (ZZ zoom) are represented by **I** number of contours, **J** contour width, **K** air gap, **L** filament width, **M** angle of deposition, and **N** layer thickness; and filament’s characteristic (WW zoom of the deposited filaments) is represented by **O** ellipsoid shape of filament.

Build parameters in FDM technology include tip size, layer thickness, raster width, raster angle, air gap, direction respect to the build bed, contour number, and contour width [66,71]. Some of these are shown in Figure 1. To build a part the filament is pulled out of the canister using rollers. The filament is taken to a pair of plates that contain heating resistors that take the material to the liquefaction state. The material then flows through a circular tip nozzle. The system has two different nozzles, one for model material and one for support material [66,71]. The material is deposited following a build strategy, which depends on the component geometry, cost, weight, inertia, and usage [8,72].

The inner structure of FDM components has a pattern caused when the material is extruded from a circular tip and deposited. The filament has a transversal ellipsoid shape caused by the shrinkage of the material. The filaments are bounded by the larger axis of the ellipse [15,65,73].

## 2. Materials and Methods 

### 2.1. Samples

**Figure 2:**
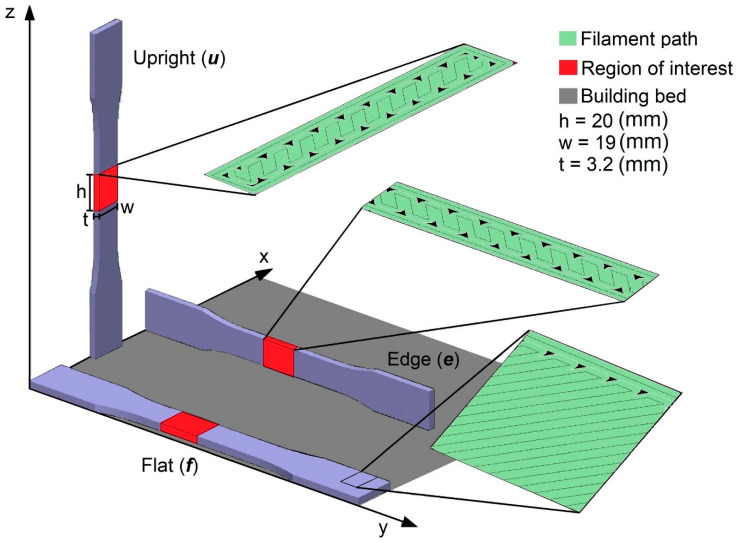
Built samples in flat, edge, and upright orientations based on ASTM D638 type 1, showing the filament path in green, regions of interest in red, with a size of 20 × 19 × 3.2 mm^3^, and building bed in dark gray.

Fifteen tensile samples, based on the standard D638 type 1 [74], five per orientation flat (***f***), edge (***e***), and upright (***u***) (see Figure 2), were manufactured by Stratasys Ltd (Eden Prairie, Minnesota) using Fortus 900 mc machine (FDM technology) and ABS-M30 material. The build parameters were a layer thickness of 0.254 mm, a raster width of 0.5080 mm, a 45°/−45° raster angle, an air gap of 0 mm, two contours, and build mode “solid”. These parameters were chosen using default parameters based on the material datasheet [75] and were also used by other authors to report mechanical properties of the technology-material combination [14,17,76].

### 2.2. CT Scan

One sample per build direction ***f***, ***e***, and ***u*** were inspected using a CT scan, Nikon XT H225ST (Nikon Metrology, Inc., Brighton, MI, USA), with the following parameters: Perkin Elmer detector of 16 bits, 2000 × 2000 pixel size, 140 kV voltage, 220 µA current, 354 ms exposure time, and a voxel resolution of 13 µm.

#### Voids Quantification

The authors of this work classify the void descriptors as global and local ones. A global descriptor describes the general void characteristics of the sample, such as the void-to-volume ratio (***Vr***), while void size and shape refer to local descriptors, a more specific characteristic associated to each void. The descriptor includes the measurement of the void size in three dimensions: 1D sphere diameter (***d***), projected size (***ps***), 2D projected area (***pa***), and 3D number of voxels (***vx***). Shape descriptors commonly reference two shape ratios: sphericity (***sp***) and compactness (***cp***).

The 3D image was reconstructed using Nikon’s proprietary software CT Pro 3D (version XT 3.1.9, Nikon Metrology, Inc., Brighton, MI, USA), with default presets and no filter applied. Void quantification was performed to a region of interest (ROI) with a dimension of 19 × 3.2 × 20 mm^3^ located at the center of the samples (Figure 2), using a porosity analysis module within the Volume Graphics (VG) Studio Max (version 3.3, Volume Graphics, Heidelberg, Germany) software, where automatic surface detection was used with a void size filter set to a minimum void size volume of 8 voxels, resulting in 2 × 2 × 2 voxels, i.e., 0.026 × 0.026 × 0.026 mm^3^. The maximum void size was set as large as the analyzed ROI, using the VG user’s manual recommendations for data quality [37].

**Figure 3:**
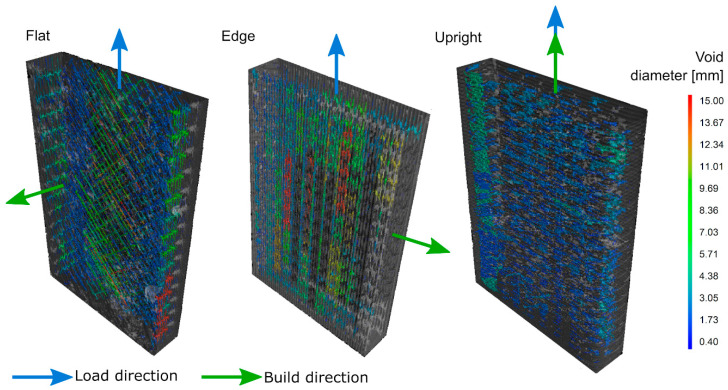
Voids visualization from CT (computed tomography) scanned samples.

In order to select the elongated voids, a filter with sphericity settings between 0 and 0.3 was applied [40,77,78,79]. The void distribution along the ROI for each built direction is shown in Figure 3. The resulting data from the filtration process contain information about global and local descriptors: ***Vr***, ***d***, ***ps***, ***pa***, ***vx***, ***sp***, and ***cp***. The quantitative mean and standard deviation of these data are presented in Table 1.

The void-to-volume ratio, sphericity, and compactness descriptors do not show an evident standalone variation according to the change in mechanical performance, although they might be referenced for filtering conditions [52,55,60,63]. The orientation within the three samples indicates that the evaluated voids are similar in shape, as expected from the filtration process.

From the 3D size descriptor, ***vx***, it is possible to note that the highest mean values belong to ***f*** and ***e*** samples, while the lowest values are for ***u***. These results, along with the low variation in ***Vr***, resemble other reports in the area of investigation, indicating no obvious correlation between void characteristics and the mechanical performance of the samples [57,59]. However, the sizes measured in ***ps*** and ***pa*** show that the voids in the three samples have a different growth direction or orientation. This difference is clearer in the 1D measure. The mean value for 1D in ***e*** shows that their voids grow in the z-direction, y- and z-directions for ***f***, and the y-direction for ***u***. In all three cases the *z*-axis is the applied load axis.

## 3. Results

### 3.1. Tensile Test Results

**Figure 4:**
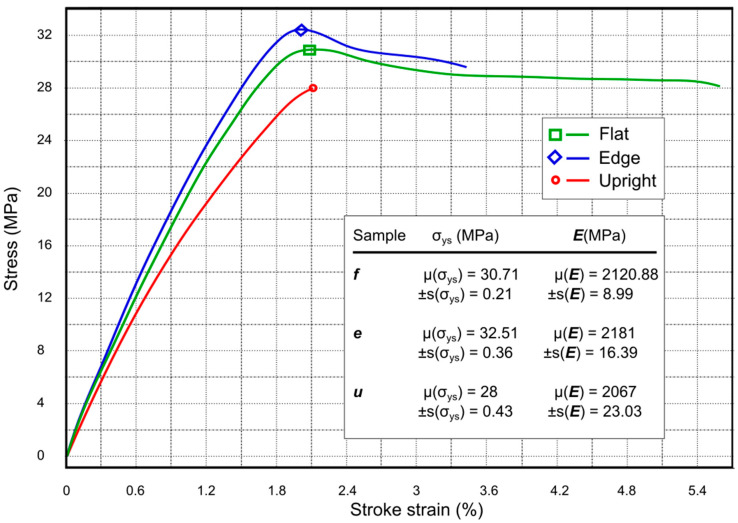
Evaluated strain–stress curve of the samples of ***f***, ***e***, and ***u***. Experimental results of mean (μ) and standard deviation (***s***) of yield strength σ_ys_ and Young’s modulus ***E***.

**Figure 5:**
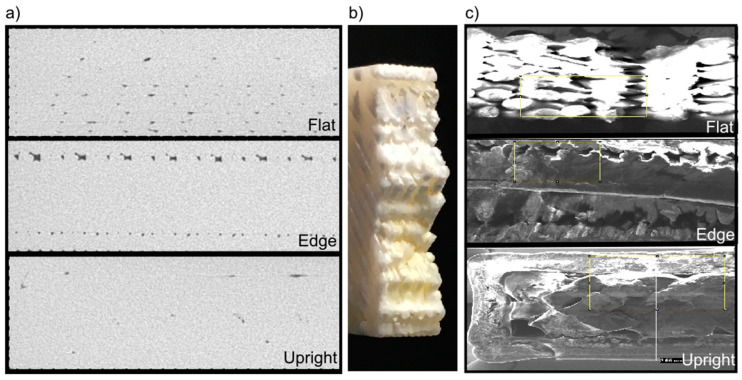
Transversal view of samples (**a**) ***f***, ***e***, and ***u*** of CT images before tensile test, (**b**) photo of the fracture on ***f*** sample, and (**c**) fracture on ***f***, ***e***, and ***u*** samples using electronic microscope.

The yield strength (σ_ys_) and Young’s modulus (***E***) of the test samples were performed using a Shimadzu AGS-X-50 kN universal testing machine (Kyoto, Japan), with a load cell of 50 kN. According to the standard ASTM D638 type 1 [74], five pieces of each orientation were tested using a velocity of 5 mm/min. The stress–strain curve was measured and generated by the software TrapeziumX by Shimadzu (Kyoto, Japan). The results for the mean and standard deviation values are displayed in Figure 4. In Figure 5a a sectional view by CT of the samples before the tensile test is shown; additionally, a view after the tensile test using an Electronic Microscope Philips XL20 (Philips, Amsterdam, The Netherlands) at 20 (KV) and a zoom of 23× is shown in Figure 5c.

Mechanical tests results show that the σ_ys_ mean value for ***e*** orientation is 5.53% and 13.87% higher than ***f*** and ***u***, respectively. The same tendency is shown for the ***E*** mean values with 2.75% and 5.22% higher also than ***f*** and ***u***. These results agree with other authors [13,75,80], where mechanical performance in the ***e*** orientation is higher than ***f*** and ***u***.

### 3.2. Void Analysis

According to Table 1, there is no direct equivalency between the digital measurements, with respect to a single descriptor data, and the mechanical properties. However, some descriptors meet the order of strength with the measured values, i.e., ***e*** is more resistant to the applied force than ***f***, which again is more resistant than ***u***. The descriptor that meets this tendency is the 1D digital measurement, ***ps***. This descriptor is suitable to be used as an approximation of a digital measure that indicates the potential mechanical resistance.

The directionality of the void elongation has an impact on the strength of the component, as reported in other publications [25,50,64]. This observation can be related with a new descriptor based on the directionality of the voids to establish a relation with the mechanical properties.

**Figure 6:**
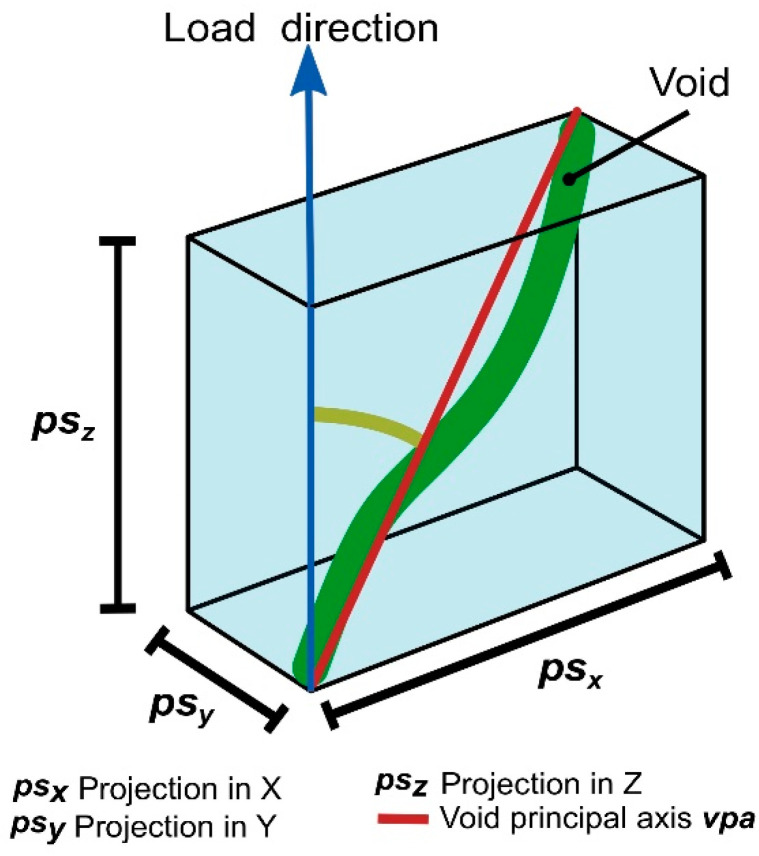
Void orientation angle α using x (***psx***), y (***psy***), and z (***psz***) void 1D projection measurements.

To define void orientation angle, which relates the void descriptors with mechanical properties, the authors of this work used 1D projection measurements provided by Volume Graphics on the three orthogonal directions x, y, and z (***psx***, ***psy***, and ***psz***, respectively) to define a rectangular prism, where the void is inscribed as is shown in Figure 6. The diagonal connecting opposite vertices represents the void principal axis, ***vpa***.

The resulting angle represents the void orientation, α, which is the angle between the load axis and ***vpa***. This angle was calculated using the following trigonometric Equation:

(1) $$tan\left(\alpha \right)=\frac{\sqrt{{\left(psx\right)}^{2}+{\left(psy\right)}^{2}}}{psz}$$

The mean and standard deviation values of the applied equation are shown in Table 2.

**Figure 7:**
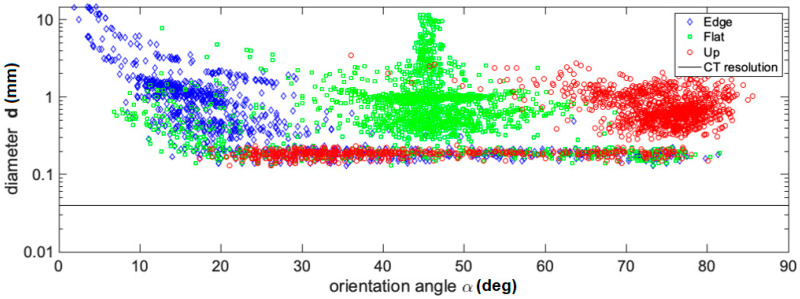
Void size (***d***) and orientation angle (α) relation. The black line indicates the CT resolution limit due to the used parameters and sample size.

Figure 7 shows a comparison of the void’s orientation angle, α, with the void diameter, ***d***, as well as with the sphericity, ***sp***, of every void. It is possible to identify from Figure 7 that maxima, minima, mean, and variance values of void descriptor measurements coincide with the results of Table 1. The parameter α in Table 2 gives a clear classification of void characteristics. Moreover, the mechanical strengths in inverse order (see Figure 4) are congruent with μ(α) and Equation (1).

### 3.3. Sensibility and Specificity

Voids with an orientation angle in the range {α:α > μ(α) + ***s***(α)} or {α:α < μ(α) − ***s***(α)} according to the corresponding build direction are likely misrelated to a distinct build direction. To validate the mean void orientation angle range for every sample, the sensitivity λ [81] is calculated for the established range. The value of λ is a statistical measure that gives the percentage of void angles correctly identified within the established angle range. For every orientation, ***e***, ***f*,** and ***u***, there is a collection **A**(α) of measured void angles with mean μ(α) and s(α) standard deviation corresponding to that orientation, and **A_¬_**(α) (w.r.t. μ**_¬_**(α),s**_¬_**(α)) and **A^¬^**(α) (w.r.t. μ**^¬^**(α),s**^¬^**(α)) corresponding to the other two collections. To calculate λ, the following Equation is used:

(2) $$\lambda =\frac{\left|TP\right|}{\left|TP\right|\;+\;\left|FN\right|}$$

where true positives (*TP*) are the voids, characterized by **A**(**α**), within the established range, and false negatives (*FN*) are voids out of this class within the range:

TP = {α′ ∈ A(α): |α′−μ(α)| < s(α)}

$$FN\;=\;\{\;\alpha \prime \;\in \;A_{\neg }(\alpha )\;\cup \;A^{\neg }(\;\alpha ):\left|\alpha \prime -\mu \left(\alpha \right)\right|\;<\;s\left(\alpha \right)\}$$

As a complement of the λ value, the specificity (ε) [81] is calculated for the range μ(α) ± ***s***(α). The specificity is also a statistical measurement—in this case, ε represents the percentage of void angles not included in the sample range **A**(α) that are correctly not contained within the range—and belongs to another void angle range. To calculate ε with respect to **A**(α), the following Equation is applied:

(3) $$\varepsilon =\frac{\left|TN\right|}{\left|TN\right|\;+\;\left|FP\right|}$$

where true negatives (TN) are the voids classified by **A_¬_**(α) and **A^¬^**(α) that are not contained in the designated angle range, and false positives (FP) are the voids of **A**(α) beyond the expected range of void angles:

$$TN\;=\;\{\alpha \prime \;\in \;A_{\neg }(\alpha )\;\cup \;A^{\neg }(\alpha ):\;\left|\alpha \prime -\mu \left(\alpha \right)\right|\;\geq \;s\left(\alpha \right)\}$$

FP ={α′ ∈ A(α) : |α′−μ(α)| ≥ s(α)}

**Figure 8:**
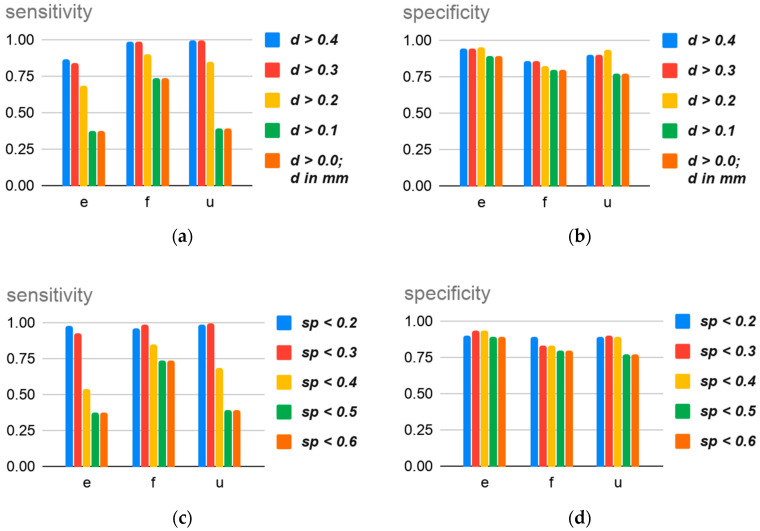
Sensitivity and specificity calculated for an angle range of |α − μ(α)| < ***s***(α) of minimum ***d*** (**a**,**b**) and maximum ***sp*** (**c**,**d**), for each collections of voids by built direction.

The results for λ and ε with reference to the range {|α − μ(α)| < ***s***(α)} evaluated with respect to a minimum diameter and a maximum sphericity are shown in Figure 8. From this void quality analysis, it is possible to observe a higher reliability for voids with a bigger orientation angle. However, collections of voids of small diameters (***d*** ≤ 0.2 mm) bring noise into the characterization of voids by their orientation angle. The authors identify that a diameter of ***d*** = 0.2 mm provides average values of λ = 81.3% and ε = 90.6%. Moreover, the sphericity of 0.3 provides a better threshold for voids in this case study, since it provides average values of λ = 97.45% and ε = 89.0%. However, it is necessary to review diameter and sphericity values after the application of the method to other materials and technologies.

**Figure 9:**
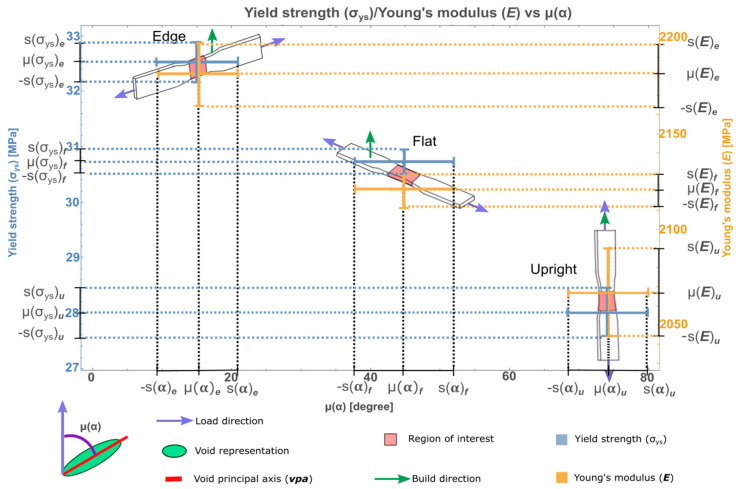
Void orientation angle μ(α) and its effect upon yield strength (σ_ys_) and Young’s modulus (***E***).

Once λ and ε were calculated, the relation between α distribution and the mechanical properties (yield strength and Young’s modulus) could be shown, as in Figure 9. This plot shows the angle μ(α) range proposed for each sample in the *x*-axis. The right ordinate represents the Young’s modulus, ***E***, which increases when the value of μ(α) is lower. In the left ordinate axis the yield strength is placed, σ_ys_ which, as well as ***E***, increases when the value of μ(**α**) is the lowest. The ranges are represented by crosses, yellow for ***E*** and blue for σ_ys_, where mean values represent the center and are delimited for one positive and one negative standard deviation, since both values of ***E*** and σ_ys_ are close to each other between samples.

Graphical results which relate mechanical prediction based on the void orientation angle and angle range are set using the calculated mean value, μ(α), plus one positive and one negative standard deviation for every sample (***e***,***f***,***u***). The resulting mean orientation angle ranges {α:|α − μ(α)| < ***s***(α)} are 9.33 to 20.93 (degrees), 37.73 to 51.90 (degrees), and 66.90 to 79.61 (degrees), for ***e***, ***f***, and ***u***, respectively. These ranges define a well-established angle region for every sample, which corresponds to the sample’s mechanical properties. The use of two or more standard deviations may cause an overlapping of the angle ranges. This graph also contains the void size filter of 0.026 × 0.026 × 0.026 mm^3^ and the shape range from 0 to 0.3.

The obtained results can be applied in AM FDM/ME to predict mechanical performance by performing a CT scan of the component, calculating the mean and standard deviation of void orientation angle and comparing the results with Figure 9. If the resulting mean value is closest to zero degrees, i.e., the voids are parallel to the load direction, then the mechanical performance will be around 81.03% for σ_ys_ and 86.13% for ***E*** of the value of an injected sample. Otherwise, if the mean value is close to 90 degrees, i.e., the voids are perpendicular to the load direction, then the mechanical properties can be reduced until around 69.79% for σ_ys_ and 81.62% for ***E*** of an injected sample.

## 4. Discussion

The results presented in this work show that descriptors like void-to-volume ratio, diameter, number of voxels, sphericity, and compactness have no equivalent relation with mechanical properties as mentioned by Lambert et al. [57]. However, the proposed void orientation angle descriptor, μ(α), shows a relation with the mechanical properties of yield strength and Young’s modulus, obtained during tensile tests for FDM components. From the plot in Figure 9, it is possible to predict the mechanical properties of components after their construction based on the calculation of its void orientation of the real structure against some finite element analysis (FEA) [82,83]. The descriptor proposed in this work is also a simplification of other methods that combine 3D imaging obtained by CT and FEA [84,85]. However, it remains unsure to what extent the measured mean values are likely to repeat, if AM process adjustments are taken.

The average void orientation angle for FDM components, μ(α), defines the growing direction of voids along the internal structure of each part. Since these voids grow following a direction and become elongated, the authors of this work propose that the angle μ(α) can be considered as a local descriptor, along with sphericity and compactness.

The proposed void orientation angle descriptor, under the given circumstances, highlights which void measurements are following the performance of mechanical properties for FDM technology with ABS material, which could be extended for other filament materials and technologies, in this case the void orientation angle; the closer μ(α) is towards 0 degree, the stronger the part. However, the proposal of this work is the first approach to develop an extended method to predict yield strength and Young’s modulus values for the analyzed part under general AM processes. Furthermore, it seems that yield strength is more closely related to void orientation angle than Young’s modulus, but more tests are required.

The results show that the measured mechanical properties, σ_ys_ and ***E***, relate to the mean void orientation (μ(α)) about the load axis: the more parallel (close to 90°) the stronger the part, as other authors refer to parts with elongated voids with different materials [86,87,88,89,90].

It remains unclear whether the orientation angle maintains its importance within distinct AM processes. It is likely that AM processes that hold issues of material fusion, like ME/FDM, result in oriented voids. Due to the definition of the void orientation with respect to void projection in x, y, and z axes, it becomes obvious that, although mathematically well established, in the case of round pores the physical implementation is vague (~45°). For this reason, the application of the void orientation angle descriptor is suggested for parts containing elongated voids.

## 5. Conclusions

In this research five samples in three different directions: flat, edge, and upright were built by ME/FDM technology and tensile tested. One of every build orientation was scanned using CT to quantify and analyze voids, including their relation with the mechanical properties of yield strength σ_ys_ and Young’s modulus ***E***.

It was found that the descriptor size in 1 dimension shows that the orientation of the void for the load direction is different for each sample. To identify void orientation a new descriptor was proposed, α, the angle between the principal void axis, ***vpa***, and the load direction, using μ(α); a range of values was set to establish a new criterion.

The proposed descriptor, μ(α), shows that the void’s ***vpa*** oriented parallel to the load axis increase yield strength and Young’s modulus unlike the perpendicular voids. From these results, it is possible to estimate the mechanical performance of FDM samples via CT and comparing void orientations with the plot developed in Figure 9.

This work develops and applies a descriptor focusing on void angles for predicting the mechanical properties of AM parts using NDT. However, the criteria developed in this work have only been tested on ME, and further experimentation with different processes, materials, and build orientations are required.

## Figures and Tables

**Table 1:** Defect detection mean (μ) and standard deviation (***s***) of void characteristics for tensile samples.

Characteristic	Descriptor	μ and *s*	Flat	Edge	Upright
Void-to-volume ratio	*Vr* (%)	-	0.682	0.690	0.587
Size	*d* (mm)	μ(*d*)	1.604	2.105	0.992
		*s*(*d*)	1.741	2.367	0.204
	*ps* (mm)	μ(*psₓ*) μ(*ps_y_*) μ(*ps_z_*)	0.210 1.137 1.202	0.254 0.338 2.099	0.440 0.854 0.331
		*s*(*psₓ*) *s*(*ps_y_*) *s*(*ps_z_*)	0.198 1.246 1.247	0.073 0.228 2.367	0.350 0.400 0.273
	*pa* (mm^2^)	μ(*pa_xy_*) μ(*pa_xz_*)	0.100 0.113	0.049 0.250	0.121 0.077
		*s*(*pa_xy_*) *s*(*pa_xz_*)	0.119 0.158	0.050 0.382	0.102 0.088
	*vx*	μ(*vx*)	3483.521	9412.199	5049.888
		*s*(*vx*)	5309.964	20,932.911	8606.976
Shape	*sp*	μ(*sp*)	0.239	0.241	0.246
		*s*(*sp*)	0.043	0.048	0.043
	*cp*	μ(*cp*)	0.008	0.0048	0.015
		*s*(*cp*)	0.006	0.005	0.007

**Table 2:** Mean (μ) and standard deviation (***s***) of void orientation angle according to build direction.

Characteristic	Descriptor		Flat	Edge	Upright
Orientation	Angle α (degree) between the principal void axis and the load axis	μ	44.822	15.134	73.257
		*s*	7.084	5.801	6.355
